# Effect of risk-based payment model on caries inequalities in preschool children assessed by geo-mapping

**DOI:** 10.1186/s12903-017-0470-6

**Published:** 2018-01-05

**Authors:** Anders Holmén, Ulf Strömberg, Gunnel Håkansson, Svante Twetman

**Affiliations:** 1 Department of Research and Development, Halland Hospital, 302 33 Halmstad, Sweden; 2 Institute of Medicine, Health Metrics Unit, Sahlgrenska Academy at University of Gothenburg, PO Box 463, 405 30 Gothenburg, Sweden Sweden; 3 Public Dental Health Unit, Region Halland, PO Box 517, 301 80 Halmstad, Sweden; 4 Department of Odontology, Faculty of Health and Medical Sciences, University of Copenhagen, Nørre Allé 20, Copenhagen, Denmark

**Keywords:** Caries, Children, Prevention, Geo-mapping, Time trend

## Abstract

**Background:**

To describe, with aid of geo-mapping, the effects of a risk-based capitation model linked to caries-preventive guidelines on the polarization of caries in preschool children living in the Halland region of Sweden.

**Methods:**

The new capitation model was implemented in 2013 in which more money was allocated to Public Dental Clinics surrounded by administrative parishes inhabited by children with increased caries risk, while a reduced capitation was allocated to those clinics with a low burden of high risk children. Regional geo-maps of caries risk based on caries prevalence, level of education and the families purchasing power were produced for 3-6-year-old children in 2010 (*n* = 10,583) and 2016 (*n* = 7574). Newly migrated children to the region (*n* = 344 in 2010 and *n* = 522 in 2016) were analyzed separately. A regional caries polarization index was calculated as the ratio between the maximum and minimum estimates of caries frequency on parish-level, based on a Bayesian hierarchical mapping model.

**Results:**

Overall, the total caries prevalence (dmfs > 0) remained unchanged from 2010 (10.6%) to 2016 (10.5%). However, the polarization index decreased from 7.0 in 2010 to 5.6 in 2016. Newly arrived children born outside Sweden had around four times higher caries prevalence than their Swedish-born peers.

**Conclusions:**

A risk-based capitation model could reduce the socio-economic inequalities in dental caries among preschool children living in Sweden. Although updated evidence-based caries-preventive guidelines were released, the total prevalence of caries on dentin surface level was unaffected 4 years after the implementation.

**Electronic supplementary material:**

The online version of this article (10.1186/s12903-017-0470-6) contains supplementary material, which is available to authorized users.

## Background

Dental caries is one of the most common chronic conditions worldwide with more than 3 billion people affected [1, 2]. It is also evident that dental caries, like most other lifestyle-related non-communicable diseases, is unevenly distributed in the population with a clear socioeconomic gradient [3, 4]. In order to inform the regional public health authorities, we previously illustrated the inequalities in caries risk among children living in Region Halland in southwest Sweden with aid of a geo-mapping tool [5]. It was also shown that this tool could be used to unveil time trends in childhood caries risk, and thereby offer a possibility to monitor and assess the outcome of preventive programs [6]. We observed an increasing polarization of caries risk in the Halland region between 2006 and 2010 and this was the driving force for a new payment model to the Public Dental Clinics in the region.

With the ambition to address and reduce the socioeconomic gaps in caries risk, the regional dental health authorities decided in 2012 to change the payment model and allocate the monetary resources to the different local Public Dental Clinics according an algorithm that was developed from the estimated caries risk (Table 1). The region has approximately 300,000 inhabitants and the vast majority of all children are listed as regular patients at the Public Dental Service that provides free dental care between 1 and 20 years through a capitation system. With the new model, dental clinics with a higher burden of high risk children were allocated an increased amount of money per child and year, on expense on those clinics with children with lower risk that received a reduced annual fee per child. Linked to this re-allocation of resources, the regional strategies for risk-based caries prevention were updated according to best available evidence. The new guidelines were implemented at the dental clinics in 2013. In brief, for healthy children with no signs of active early or moderate lesions, twice daily tooth brushing with fluoride toothpaste was the cornerstone while children with active caries progression and/or medical and social risk factors were supplied by repeated professional fluoride varnish applications and fissure sealants. Children with the highest caries risk were provided with individually targeted measures after motivational interviewing. The aim of the present study was, with aid of the geo-mapping tool, to evaluate and describe the effects of the novel capitation and risk-based strategies on caries prevalence, 4 years after the implementation. In this report, we focus on preschool children 3-6 years of age and it was thought of special interest to analyze whether or not the inequalities in caries risk had been reduced.

**Table 1:** Variables of the algorithm for calculating the risk-based allocation of annual capitation on administrative parish-level (i.e., the same amount of money was allocated for each child living in a parish)^a^

Parish-level variable^b^	Score	Proportion in each category, overall
Level of higher education (%)	1: >24.6%	33.3%
	2: 17.7-24.6%	33.3%
	3: <17.7%	33.3%
Non-Swedish background (%)	1: ≤7%	33.3%
	2: 7.1-9.5%	33.3%
	3: >9.5%	33.3%
Low purchasing power (<135,000 SEK; %)	1: ≤17.9%	33.3%
	2: 18.0-25.0%	33.3%
	3:>25.0%	33.3%
Calculated caries risk	1: ≤0.60	20%
	2: 0.61-1.33	60%
	3:>1.33	20%

^a^ Algorithm is based on a total score for children between 3 and 6 years: Total score = 0.2*education_score + 0.2*foreign background_score + 0.2*purchase power_2 + 0.4*caries risk (Maximum score = 3.0; minimum score = 1.0)

^b^ Statistics Sweden provided parish-level data on three socio-economic indicators: i) the proportion with post-secondary education among all residents; ii) the proportion of immigrants (more specifically, individuals born outside Sweden and individuals born in Sweden with both parents born outside Sweden) among all residents; and iii) the proportion of families with low purchasing power (according to Swedish standard) among all residing families with at least one child (<20 years old; family with the same residence address)

## Methods

### Study groups

The material consisted of 10,927 children, 3-6 years of age and born in Sweden, that were examined 2010 and 8096 comparable children examined in 2016. Children that had migrated to the region from countries outside Sweden, mainly from the Middle East, during the study period were analyzed separately. These children constituted 3.1% (*n* = 344) of the entire study population in 2010 and 6.4% (*n* = 522) in 2016. The study was approved by the Halland Hospital Ethical committee as well as The Swedish Data Inspection Board.

### Caries data collection

The experience of manifest (dentin) caries, scored according to the WHO-criteria [7], was collected from the regional epidemiological database. This database contains caries data from all children 3-19 years attending the Public Dental Service in the Halland region. The children were examined by the regular responsible dentist and the scores were filed into digital records. All dentists were regularly trained and calibrated within the clinic at least once every second year. The frequency of clinical examinations for children aged 3-6 years changed over the study period from yearly to less frequent since the new guidelines implemented in 2013 allowed extended recall intervals for children assessed with low caries risk (Additional file 1).

### Geo-mapping and statistical methods

The Halland region consists of six municipalities that are subdivided into 58 administrative parishes. Each child was geo-coded with respect to his/her residence parish. Geo-maps were produced by using the ESRI® ArcGIS system (Environmental Systems Research Institute, Inc., USA). A parish-level relative risk (RR) was calculated as the observed-to-expected ratio, where the expected number was obtained from the sex-specific caries (dmfs > 0) rates for the total study population of 3-6 year old children residing in the Halland region. Moreover, smoothed RRs (SmRR) for each administrative parish were obtained by running a Bayesian hierarchical mapping model, which allow parish-specific RRs to be smoothed towards global and local average risk levels across the study region [8]. We underline that such Bayesian smoothing yielded “shrinkage” of the conventional observed-to-expected ratios. The corresponding statistical certainty geo-maps were obtained by calculating the posterior probabilities of a parish-specific relative risks above 1 given the data, denoted Pr (RR > 1|data), using the Bayesian approach. A parish with data yielding strong statistical evidence of an elevated caries risk (Pr (RR > 1|data) >0.95) was colored red in the certainty geo-map. By contrast, a parish with evidently lowered caries risk (Pr (RR < 1|data) = 1 – Pr (RR > 1|data) > 0.95) was colored green. The remaining parishes were colored yellow, which indicates a weaker statistical evidence for an elevated/lowered relative risk.

**Fig. 1:**
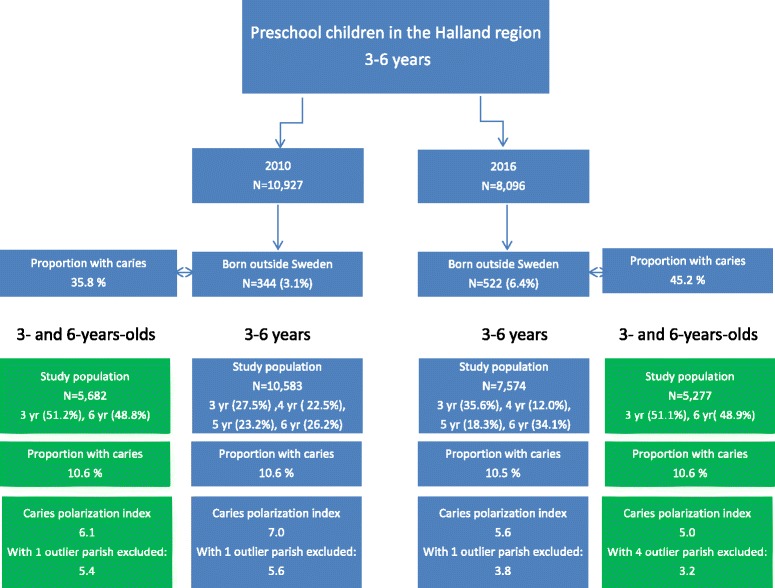
Study flowchart with age distribution, caries prevalence and caries polarization index

In the present report we summarize the spatial risk pattern by the *regional* polarization of caries risk. A caries polarization index for a specific calendar year (2010, 2016) was calculated as the ratio between the maximum and minimum estimates of relative risk. We also performed a sensitivity analysis of the polarization index by excluding “outlying” administrative parishes. An administrative parish was regarded as an “outlier” if the estimated RR of caries for the children living in that parish corresponded to an outlying (or extreme) value according to a conventional statistical definition based on the box-plot of the estimated RRs (IBM SPSS Statistics for Windows, Armonk, NY). Moreover, the polarization index was re-calculated by excluding the 4-5-year-olds, who were examined less frequently in 2016 (Fig. 1).

The statistical computations were performed using SPSS (version 24.0) and the free software Rapid Inquiry Facility [9], which provides an extension to ESRI® ArcGIS functions [10], along with free software for Bayesian data analyses, WinBUGS [11].

## Results

**Fig. 2:**
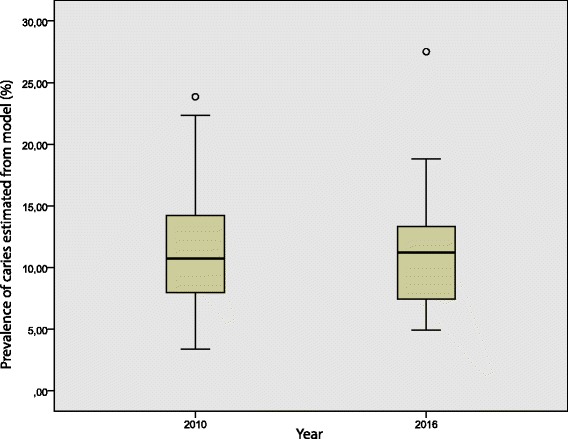
Box-plots of parish-specific prevalence of caries (58 parishes) estimated from a Bayesian hierarchical mapping model. Data from 3 to 6-year old children born in Sweden living in the Halland region, examined in 2010 and 2016, respectively. An open circle indicates a parish with statistically outlying caries prevalence

**Fig. 3:**
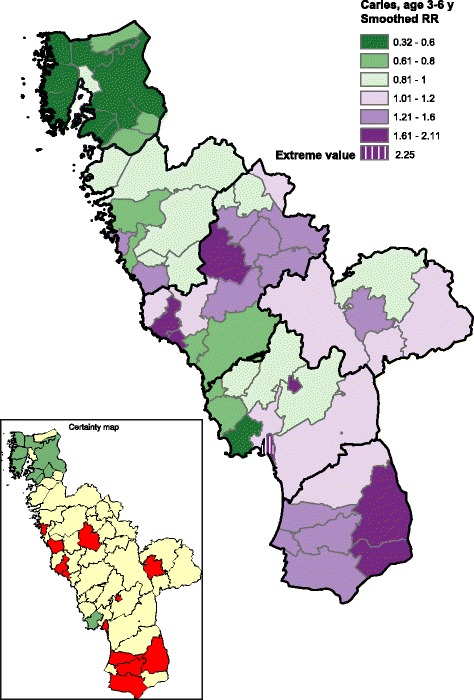
Geo-map of caries risk in 3-6-year old children born in Sweden and living in the Halland region, examined in 2010. The map displays, for each of the 58 residential parishes, the smoothed relative risk (SmRR, range between 0.32-2.25) of caries (dmfs > 0) among preschool children (3-6 years. The corresponding statistical certainty geo-map is also shown [*red color*, Pr (RR > 1|data) >0.95, i.e. a parish with data yielding strong statistical evidence of an elevated caries risk; *green color*, Pr (RR < 1|data) >0.95, i.e. a parish with data yielding strong statistical evidence of a low caries risk; and *yellow color*, the 90% credibility interval covers RR = 1, i.e. a parish with data yielding weaker statistical evidence for a high or low relative risk]

**Fig. 4:**
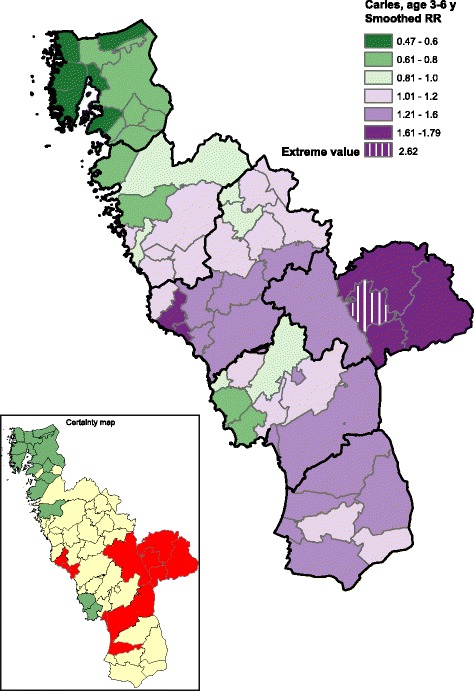
Geo-map of caries risk in 3-6-year old children born in Sweden and living in the Halland region, examined in 2016. The map displays, for each of the 58 residential parishes, the smoothed relative risk (SmRR, range between 0.47-2.62) of caries (dmfs > 0) among preschool children (3-6 years). The corresponding statistical certainty geo-map is also shown [*red color*, Pr (RR > 1|data) >0.95, i.e. a parish with data yielding strong statistical evidence of an elevated caries risk; *green color*, Pr (RR < 1|data) >0.95, i.e. a parish with data yielding strong statistical evidence of a low caries risk; and *yellow color*, the 90% credibility interval covers RR = 1, i.e. a parish with data yielding weaker statistical evidence for a high or low relative risk]

A flowchart showing the age distribution, caries prevalence and caries polarization indices is presented in Fig. 1. Due to the marked differences in age distribution in the groups between 2010 and 2016, results are presented for the entire study groups (3-6 year) as well as for the merged 3- and 6-year-olds. The excluded children born outside Sweden had significantly higher caries prevalence than the Swedish-born children, both in 2010 (35.8% vs. 10.6%, *p* < 0.001) and in 2016 (45.2% vs. 10.6%, p < 0.001). Although the caries prevalence among the 3-6 year old children remained the same (10.6% in 2010 and 10.5% in 2016), the caries polarization index was markedly lower in 2016 (5.6) than in 2010 (7.0). When parishes with statistically outlying RRs were excluded (*n* = 1 in 2010 and n = 1 in 2016; Fig. 2), the corresponding values became 3.8 and 5.6, respectively. The geo-maps from 2010 and 2016 for 3-6-year old children are presented in Figs. 3 and 4. It can be clearly seen that the caries-risk map did change over the years; out of the five parishes with the highest caries risk in 2010, four were lowered in 2016. On the other hand, the less densely populated inland parishes to the east exhibited an increased caries risk. When repeating the analysis for the merged 3- and 6-year-olds (i.e., excluding the 4-5-year-olds), the caries polarization indices deceased somewhat; yet, lower values were observed in 2016 (Fig. 1).

## Discussion

The need to reduce social inequalities in general and oral health is commonly addressed [12] and it has been suggested that the dental team can play an important role in these efforts [13]. In this context, the common risk factor approach has been highlighted, integrating caries prevention with the prevention of other life-style related chronic diseases such as the metabolic syndrome [14]. In our previous report, we observed an increasing polarization of caries risk in the Halland region between 2006 and 2010 [6] and this was the driving force for a new payment model to the Public Dental Clinics in the region. The money were allocated based on the actual caries risk in administrative perishes surrounding the clinic and the capitation was linked to a preventive program. The main result of the present study was that the trend of increasing polarization seemed to be terminated. In fact, our present data indicated a reduced polarization of caries among the 3 to 6-year-olds in the region. This was an important finding in the light of another recent report from Scandinavia concluding that the social gaps in caries occurrence unfortunately is increasing among adolescents [15]. It is tempting to believe that this positive trend was due to the new risk-based payment model but there are a number of other factors that could have played a role. A confounding factor was the high amount of immigrant and refugee families that migrated to Sweden during the study period, the majority between 2015 and 2016. Since their children only had the opportunity to benefit from the Public Dental Service system for a limited time period, they were analyzed separately. A striking finding was the four times higher caries prevalence among those newly arrived preschool children and the need of urgent and comprehensive dental care for this group of children was obvious. Thus, these children meant a non-scheduled extra burden to the public dental clinics in terms of language barriers and extensive emergency treatments that may have crowded out the care of the permanent residents. Another possible bias was that the compliance with the updated preventive program may have differed between the 20 clinics belonging to the Public Dental Service in the region. The evidence-based guidelines were implemented from 2013 but it is well known that it may take a while for changed routines to be administrated and established. Unfortunately, we were unable to collect reliable and detailed information from the different clinics how the recommendations were received and to what extent they were followed. An obvious effect of the program was however seen; the age distribution within the age span 3-6 years was shifted in 2016 when compared to 2010. The explanation for this was that the new guidelines allowed prolonged follow-up intervals for children assessed as low caries risk at the 3-year examination. The background thinking was if the low-risk children were recalled less often, more time could be spent on children with an estimated higher risk. Consequently, the 2016 cohort consisted of a higher proportion of 3- and 6-year-olds children and relatively fewer children aged 4 and 5 years (Fig. 1). It is thereby possible that the focus on the children with the highest dental needs was the main reason for the reduced polarization. It should however be underlined that the program, for better or for worse, did not seem to have an impact on the overall caries prevalence on dentin level (dmfs) among the preschool children in the region.

A third possible shortcoming was that the geo-maps were based on epidemiological data limited to dentin lesions (moderate and extensive) only. This means that the true caries prevalence was underestimated. From a previous study of 3-6 year-old preschool children living in Sweden, it has been shown that early non-cavitated enamel lesions are twice as common as manifest lesions [16]. However, we do not think that an inclusion of early lesions would dramatically have changed the outcome of this study. The detection of the early enamel lesions is crucial for an accurate caries risk assessment [17] and the presence of such lesions was certainly considered by the clinicians, although not reported to the regional office. Moreover, the updated preventive program was structured to enable non-operative arrest of early lesion so they were certainly not ignored.

The geo-mapping method estimated the spatial pattern of caries risk. In the present report, we summarized the estimated risk pattern by the polarization index for the total study region. More specific spatial changes in caries RR could also be evaluated by geo-mapping methods. We have proposed a simple method to identify administrative parishes with evidential positional changes (e.g., from an elevated to a non-elevated risk area), based on comparisons of the certainty geo-maps between different calendar years [6] and this technology was applied here for the interval between 2010 and 2016. We presented certainty maps using cut-off points of Pr (RR > 1|data) >0.95 [or Pr (RR < 1|data) >0.95 for identifying administrative parishes with elevated or lowered caries risk. Such a certainty map has been shown to have high specificity, although the sensitivity of identifying a truely elevated/lowered) risk areas might be moderate [18]. Recently, Boulieri et al. [19] have proposed another method based on multiple data sources for detecting areas where time-trends have been unusual which may add an alternative picture of the childhood caries in future studies.

It has been argued that a weakness of the risk-based allocation model could be that it lacks incitement for improvements; a clinic that successfully is reducing risk among its children is “rewarded” with less money. This may very well be true but a possible way to overcome this is a result-based bonus system that can be used as a driving force for preventive efforts that actually bridge the gaps in caries prevalence in among the vulnerable groups.

## Conclusion

Within the limitations of this study, it can be concluded that a risk-based capitation system could reduce the socio-economic polarization of dental caries in preschool children living in the Halland region in Sweden. The capitation was linked to evidence-based caries-preventive measures but the total prevalence of caries on dentine level was unaffected 4 years after its implementation.

## Additional file

Additional file 1: Guidelines for risk-based caries-prevention for children and adolescents. Data detail a flowchart of recommended preventive actions and follow-up recalls based on an individual caries risk assessment, as implemented in the public dental service in the Halland region 2013. (DOCX 23 kb)

## Abbreviations

Dmfs: Decayed missed filled surfaces in primary teeth
Pr (RR): Parish specific relative risk
RR: Relative risk
SmRR: Smoothed relative risk
WHO: World Health Organization
