# Supplementary material for: Effect of risk-based payment model on caries inequalities in preschool children assessed by geo-mapping
Source: BMC Oral Health. 2018 Jan 5;18:3. doi: 10.1186/s12903-017-0470-6 (PMC5755415; doi:10.1186/s12903-017-0470-6)
Supplement: Additional file 1: — Guidelines for risk-based caries-prevention for children and adolescents. Data detail a flowchart of recommended preventive actions and follow-up recalls based on an individual caries risk assessment, as implemented in the public dental service in the Halland region 2013. (DOCX 23 kb) [file 12903_2017_470_MOESM1_ESM.docx]

**Caries preventive guidelines for children and adolescents, Region Halland**

General recommendations:

- 3-year-olds should be always be examined and risk assessed by a dentist
- Recall examinations can be performed by dental hygienists (RDH)
- Dentists should monitor the occlusal development and assess the need for orthodontic care, particularly at the age interval between 10-11 years.
- The use of tobacco should be registered from 12 years
- From age 15, the presence of periodontal pocket should be assessed with a periodontal probe around the 1st permanent molars and the upper incisors

**3-6 years**

| Risk group | Maximal recall interval | Preventive measures |
| --- | --- | --- |
| 1. **Low risk**  - No clinical signs of active lesions; ”caries free” - No progression of lesions last 2 years | 18 months | - Tooth brushing twice daily with fluoride toothpaste, performed by parents or custodians |
| 1. **Moderate risk**  - Previous history of caries; fillings or arrested lesions - Some general risk, chronic diseases, social risk and/or suboptimal parental care | 18 months | - Tooth brushing twice daily with fluoride toothpaste, performed by parents or custodians - Professional fluoride varnish applications every 6^th^ month |
| 1. **High risk**  - Active caries development; new initial or manifest lesions - Increased general risk, chronic conditions such as asthma, diabetes, feeding problems, social risk and/or suboptimal parental care | 12 months | - Tooth brushing twice daily with fluoride toothpaste, performed by parents or custodians - Professional fluoride varnish applications every 3^rd^ month - Fissure sealing of newly erupted 1^st^ permanent molars - Individually targeted supplements, such as MI, sugar reduction |

**7-11 years**

| Risk group | Maximal recall interval | Preventive measures |
| --- | --- | --- |
| 1. **Low risk**  - No detectable lesions or single minor occlusal/buccal lesions related to tooth morphology - No lesion progression in primary teeth during the last 2 years | 18 months | - Tooth brushing twice daily with fluoride toothpaste, performed or supervised by parents or custodians |
| 1. **Moderate risk**  - 1 new dentin lesion and/or 1-2 new or progressing enamel lesions - Some general risk factors, such as chronic diseases, overweight, obesity, social risk factors | 18 months | - Tooth brushing twice daily with fluoride toothpaste, performed or supervised by parents or custodians - Professional fluoride varnish applications every 6^th^ month - Fissure sealing of newly erupted 1^st^ permanent molars |
| 1. **High risk**  - ≥2 new dentin lesions and/or ≥3 new or progressing enamel lesions - Rapid caries progression in primary teeth - Increased general risk, chronic conditions such as asthma, diabetes, overweight, obesity, social risk factors - Gingivitis not associated with tooth eruption | 12 months | *Caries:*   - Tooth brushing twice daily with fluoride toothpaste, performed or supervised by parents or custodians - Professional fluoride varnish applications every 3^rd^ month - Fissure sealing of newly erupted 1^st^ permanent molars - Individual supplements after motivational interviewing. Example: fluoride mouth rinse (0.05-0.2%)   *Gingivitis*   - Individually targeted care |

**12-19 years**

| Risk group | Maximal recall interval | Preventive measures |
| --- | --- | --- |
| 1. **Low risk**  - Caries-free or single minor occlusal/buccal lesions related to tooth morphology - No lesion progression during last 2 years | 18 months | - Tooth brushing twice daily with fluoride toothpaste |
| 1. **Moderate risk**  - 1 new dentin lesion and/or 1-2 new or progressing enamel lesions - Some general risk, such as chronic diseases, social risk, tobacco use | 18 months | - Tooth brushing twice daily with fluoride toothpaste - Professional fluoride varnish applications every 6^th^ month - Fissure sealants, newly erupted molars/premolars |
| 1. **High risk**  - ≥2 new dentin lesions and/or ≥3 new or progressing enamel lesions - Increased general risk, chronic conditions such as asthma, diabetes, overweight, obesity, social risk factors, tobacco use - General gingivitis and/or periodontal pockets ≥5 mm | 12 months | *Caries:*   - Tooth brushing twice daily with fluoride toothpaste - Professional fluoride varnish applications every 3^rd^ month - Fissure sealants, newly erupted molars/premolars - Individual supplements after motivational interviewing. Example: fluoride mouth rinse (0.05-0.2% NaF); high fluoride toothpaste   *Gingivitis/periodontal pockets:*   - Individually targeted care |
